# Modeling fortification of corn masa flour with folic acid: the potential impact on exceeding the tolerable upper intake level for folic acid, NHANES 2001–2008

**DOI:** 10.3402/fnr.v57i0.19146

**Published:** 2013-01-09

**Authors:** Heather C. Hamner, Sarah C. Tinker, R.J. Berry, Joe Mulinare

**Affiliations:** National Center on Birth Defects and Developmental Disabilities, Centers for Disease Control and Prevention (CDC), Atlanta, GA, USA

**Keywords:** Folic acid, fortification, corn masa flour, tolerable upper intake level

## Abstract

**Background:**

The Institute of Medicine set a tolerable upper intake level (UL) for usual daily total folic acid intake (1,000 µg). Less than 3% of US adults currently exceed the UL.

**Objective:**

The objective of this study was to determine if folic acid fortification of corn masa flour would increase the percentage of the US population who exceed the UL.

**Design:**

We used dietary intake data from NHANES 2001–2008 to estimate the percentage of adults and children who would exceed the UL if corn masa flour were fortified at 140 µg of folic acid/100 g.

**Results:**

In 2001–2008, 2.5% of the US adult population (aged≥19 years) exceeded the UL, which could increase to 2.6% if fortification of corn masa flour occurred. With corn masa flour fortification, percentage point increases were small and not statistically significant for US adults exceeding the UL regardless of supplement use, sex, race/ethnicity, or age. Children aged 1–8 years, specifically supplement users, were the most likely to exceed their age-specific UL. With fortification of corn masa flour, there were no statistically significant increases in the percentage of US children who were exceeding their age-specific UL, and the percentage point increases were small.

**Conclusions:**

Our results suggest that fortification of corn masa flour would not significantly increase the percentage of individuals who would exceed the UL. Supplement use was the main factor related to exceeding the UL with or without fortification of corn masa flour and within all strata of sex, race/ethnicity, and age group.

## Introduction

In 1998, the Institute of Medicine (IOM) set a tolerable upper intake level (UL) for usual daily intake of synthetic folic acid of 1,000 µg for adults aged ≥19 years of age (1). The UL, which is ‘the highest level of daily nutrient intake that is likely to pose no risk of adverse health effects in almost all individuals in the specified life stage group’ is defined as the absolute value of actual usual intake of folic acid obtained from fortified foods and from vitamin supplements and is expressed as µg of folic acid/day (1); intake of metabolically distinct naturally occurring food folate is not included in the UL. In establishing the UL, the IOM limited the review of potential adverse effects to supplemental folate (1). However, the one potential adverse effect of folic acid mentioned was the potential for ‘excessive folate intake to exacerbate or precipitate neuropathy’ among individuals with vitamin B12 deficiency as described in multiple case studies that reported neurological complications when the underlying vitamin B12 deficiency was not treated appropriately and folic acid was given in high dosages (i.e.≥5,000 µg/day) for many months (1). Of the 155 cases described in the case studies, only eight well-documented cases reported dosages below 5,000 µg/day (1).

Based upon data from these case reports, the IOM set the Lowest Observed Adverse Effect Level (LOAEL) for individual consumption of folic acid at 5,000 µg/day (1). The IOM states that, when possible, the UL is based on a No Observed Adverse Effect Level (NOAEL), ‘which is the highest intake (or experimental oral dose) of a nutrient at which no adverse effects have been observed in the individuals studied’. However, because there was no dose-response relationship for folic acid, ‘data were not available with which to set a NOAEL’ and the LOAEL was used (1). Because of the potential severity of neurologic complications associated with a vitamin B12 deficiency and the inability to set a NOAEL, the IOM applied an uncertainty factor of five to derive a UL (1,000 µg/day) for adults aged 19 years and older and extrapolated downward from the adult value based on body weight to determine values for children aged 1–18 years (i.e. 300 µg for children aged 1–3 years; 400 µg for children aged 4–8 years; 600 µg for children aged 9–13 years; and 800 µg for children aged 14–18 years) (1). Although several studies are continually cited as evidence that there could be various adverse effects, specifically colorectal cancer, associated with high folic acid intake (2, 3), extensive reviews from the United Kingdom (4, 5) and Australia and New Zealand were recently conducted to assess the potential adverse effects of folic acid and the implications for folic acid fortification (6). These reviews concluded that there was no reason based on safety concerns to alter any recommendations for folic acid fortification (4–6). Currently, no established risks have been reported of high supplemental folic acid intake in children (1, 7–9) and the issue has been raised regarding the relevance of ULs for folic acid among children (10).

A previous analysis by Hamner et al. modeled the potential impact on folic acid intake if corn masa flour (an ingredient used to make corn tortillas and other products) were fortified with folic acid (11). The basis for this previous modeling exercise was that Hispanics tend to be at a higher risk for neural tube defects (NTDs) compared to non-Hispanic whites (12–14) and report lower total folic acid consumption (15–17), yet they report higher consumption of corn masa flour (18). Therefore, by fortifying corn masa flour, folic acid intake could be increased and provide an additional opportunity for a reduction in the number of NTD-affected pregnancies among Hispanics (unpublished observations; Tinker SC, Devine O, Mai C, Hamner HC, Reefhuis J, Gilboa SM, Dowling NF, Honein MA 2012) since folic acid has been shown to significantly reduce the risk of having a pregnancy affected by an NTD (19–21). This previous modeling exercise did not address the impact on the UL for folic acid. The purpose of the current analysis was to assess the potential impact fortification of corn masa flour with folic acid could have on the percentage of the US population (adults aged≥19 years of age and children 1–18 year of age) exceeding the UL for total folic acid intake. In addition, the impact on individuals who report consuming corn masa flour was also assessed. These data are needed for policy makers to understand how fortification of corn masa flour could impact the US population.

## Materials and methods

### National Health and Nutrition Examination Survey (NHANES), 2001–2008

The description of the Subjects and Methods was adapted directly from Hamner et al. (11). The analysis presented here includes four additional survey years (2005–2008) and any methodology or analytical changes as a result of these additional years are noted. NHANES is conducted using a stratified multistage probability design. This ongoing survey captures a nationally representative sample of the non-institutionalized civilian US population. Respondents participate in a household interview and a physical examination. NHANES data are released to the public in 2-year cycles and for this analysis we used data from 2001–2008. All participants in NHANES provide written informed consent.

### Modeling of folic acid intake from corn masa flour

Methods for modeling of corn masa flour fortified with folic acid have been described previously (11). Briefly, modeling entailed four main steps: (a) identification of foods that could contain corn masa flour; (b) determination of the proportion of corn masa flour per food item; (c) determination of the amount of additional folic acid that would be derived from fortification if corn masa flour were fortified at 140 µg per 100 g of corn masa flour per food item; and (d) creation of model variables with the additional folic acid intake from corn masa flour fortified with folic acid.

Approximately 103 foods reported in the NHANES 2001–2008 were identified as containing corn masa flour (Appendix 1). An international manufacturer of corn masa flour validated these foods. As was done previously (11), foods were excluded if they contained words such as ‘white flour’ or if the conventional product used was a white flour tortilla (e.g. burritos).

As described previously (11), the proportion of corn masa flour present in each food was estimated using MyPyramid Equivalents Database for the USDA Survey Food Codes, 1994–2002, version 1.0 and version 2.0 for 2003–2004. A proxy of non-wholegrains was used to estimate the amount of corn masa flour in each identified food. For the majority of foods identified and analyzed, the entire grain component was listed as non-wholegrain. For the purpose of this analysis, corn masa flour was considered a non-wholegrain based on its manufacturing process, called nixtamalization (22). There were several foods reported in 2003–2004 that were not listed in the MyPyramid Equivalents Database version 2.0 (*n*=3); for these foods, the non-whole wheat grain component was estimated using the data from MyPyramid Equivalents Database version 1.0 (2001–2002). Sample calculations for estimating the amount of corn masa flour in each food item are available in Hamner et al. (11).

Folic acid fortification of corn masa flour was modeled by adding an additional 140 µg of folic acid per 100 g of corn masa flour. For example, if food item A had 32 g of non-wholegrain, this would result in an additional 44.8 µg of folic acid added from corn masa flour (32 g of non-wholegrain × 140 µg per 100 g). The total amount of folic acid that an individual would have consumed with folic acid fortification of corn masa flour included the estimated intake from fortified corn masa flour plus the actual reported folic acid intake from foods and supplements.

### Folic acid intake from foods

Intake of folic acid from foods (e.g. breads, pastas, ready-to-eat cereals) was estimated using one 24-h dietary recall questionnaire in NHANES 2001–2002, and two 24-h dietary recall questionnaires for NHANES 2003–2008. To calculate nutrient intake from foods, the USDA Food and Nutrient Database for Dietary Studies version 1 was used for NHANES 2001–2002 (23), version 2 was used for NHANES 2003–2004 (24), version 3 was used for NHANES 2005–2006 (25), and version 4.1 was used for NHANES 2007–2008 (26).

### Folic acid intake from supplements

During each household interview in NHANES 2001–2008, the participant was asked about his or her use of dietary supplements, including single vitamins, multivitamins, minerals, herbs, and other similar nutritional substances over the past 30 days. The interviewer recorded the name of each product and matched it to a list of known products. After the survey, NHANES staff obtained label information for each reported supplement to determine the ingredients. A participant was classified as a user of a dietary supplement containing folic acid if he or she reported taking such a supplement at least one time in the past 30 days. Folic acid intake was calculated for each supplement on the basis of folic acid content, the number of days a supplement was taken, and the quantity of the supplement taken per day during the past 30 days. The average daily folic acid intake for each supplement was calculated and the values were totaled across all supplements taken by each participant to yield the average daily amount of supplemental folic acid consumed (15). This estimate was added to the amount of folic acid consumed from foods for each day of intake for each individual.

### Analytic sample

We excluded individuals who were pregnant and those whose dietary interview did not meet minimum required standards for data quality (27). Pregnant women were excluded because many of these women report consuming prenatal vitamins, which contain 800–1,000 µg of folic acid. Among the total US population sampled (*n*=39,832), women were excluded if they reported being pregnant at the time of the survey (*n*=1,048). Individuals were also excluded if they did not meet the minimum data quality standard for dietary recall on day 1 (2001–2008) or day 2 of their dietary recall (2003–2008 only) (*n*=5,839) because of incomplete dietary records or the inability to calculate total caloric intake because of consumption of breast milk. Additional individuals were excluded because they were missing information on supplement use (*n*=353) or had reported implausibly high folic acid intake from supplements≥93 mg (*n*=2). This left a final sample size of 32,590 (82% of the eligible sample). Analyses reported by race/ethnicity were restricted to non-Hispanic white, non-Hispanic black, and Mexican American respondents because of the small number of individuals of other racial and ethnic groups. However, all race/ethnicities are included in analyses not stratified by race/ethnicity.

### Statistical analysis

Analyses were conducted using usual daily total folic acid intake with and without the modeled addition of folic acid from corn flour fortification (referred to as ‘current’ and ‘modeled’, respectively, in the presentation of results) to estimate the potential contribution fortified corn masa flour could have on total folic acid intake. It has been reported that estimates of nutrient intake based on one day of intake do not adequately account for within-person variation (28, 29). Therefore, to estimate usual daily nutrient intake we used PC-SIDE version 1.02 (Iowa State University, Ames, IA), which takes into account both between and within person variation when a subsample of the population has two or more days worth of intake, as does NHANES 2001–2008. Respondents in the 2001–2002 cycle provided only one day of intake information whereas almost all of those in the 2003–2008 cycles provided two independent days of intake information. We assumed that the day-to-day variability in intake did not change between 2001–2002 and 2003–2008 and used the within-person variance estimate based on 2003–2008 information to adjust daily intake from the entire 8-year combined sample. Usual nutrient intakes were adjusted for intake day of the week and interview method (in person vs. telephone). In calculating usual total folic acid intake, folic acid intake from supplements was added to foods and then usual intake was estimated using PC-SIDE. Detailed descriptions of this method are given elsewhere (28, 29).

We used PC-SIDE to calculate the percentage of total folic acid intake above specific cut-points (i.e. 1,000 µg or other age-specific ULs,) for the total US population and among corn masa flour consumers, and further stratified by sex, race/ethnicity (non-Hispanic white, non-Hispanic black, and Mexican American), and age groups (1–3 years, 4–8 years, 9–13 years, 14–18 years, 19–30 years, 31–50 years, 51–70 years, >70 years). Differences were tested using 2-tailed *t*-tests.

SPSS Complex Samples Design version 18.0 (SPSS Inc., Chicago, IL) was used to account for the complex survey design and to calculate all means, frequencies, *t*-tests, and Chi-square tests. All analyses were conducted using 8-year dietary weights calculated from day 1 dietary weights for the period 2001–2002 and day 2 dietary weights for the period 2003–2008, as recommended by the National Center for Health Statistics at the Centers for Disease Control and Prevention (27). For analyses conducted with PC-SIDE, standard errors were calculated using a set of 122 jackknife replicate weights. Replicate weights were calculated using a combination of day 1 dietary weights for NHANES 2001–2002 data and day 2 dietary weights for NHANES 2003–2008.

## Results

Table 1 provides demographic characteristics of the analytic sample. Sixty-nine percent (69.2%) of the sample reported being non-Hispanic white (95% CI: 65.9%, 72.4%) while 12.1% (95% CI: 10.3%, 14.2%) reported being non-Hispanic black and 9.2% (95% CI: 7.8%, 10.9%) reported being Mexican American. Over a third of the population reported consuming a dietary supplement containing folic acid (34.2%, 95% CI: 32.7%, 35.6%). Non-Hispanic whites were significantly more likely to report consuming a supplement containing folic acid as compared to non-Hispanic blacks and Mexican Americans (39.5%, 20.4%, and 19.2%, respectively, *P*< 0.05). Adults aged 31 years and older were more likely to report consuming a supplement containing folic acid than those in younger age groups. Mexican Americans were significantly more likely to report consuming corn masa flour on either day 1 or day 2 of the survey than either non-Hispanic whites or non-Hispanic blacks (64.6%, 24.7%, and 25.0%, respectively, *P*<0.05). Percentages are weighted.

**Table 1:** Demographic characteristics of the total US population and stratified by corn masa flour consumption and folic acid supplement use, NHANES 2001–2008

	Total US population		Corn masa flour consumers		Supplement non-users		Supplement users	
				
	Unweighted *n*	% (95% CI)^a^	Unweighted *n*	% (95% CI)^b^	Unweighted *n*	% (95% CI)^b^	Unweighted *n*	% (95% CI)^b^
Total	32,590	–	10,961	28.7 (27.3, 30.1)	24,052	65.8 (64.4, 67.3)	8,538	34.2 (32.7, 35.6)
Sex								
Males	16,328	48.9 (48.1, 49.7)	5,476	29.6 (28.0, 31.3)	12,355	69.0 (67.5, 70.5)*	3,973	31.0 (29.5, 32.5)*
Females	16,262	51.1 (50.3, 51.9)	5,485	27.9 (26.3, 29.5)	11,697	62.8 (60.9, 64.7)	4,565	37.2 (35.3, 39.1)
Race/ethnicity^c^								
Non-Hispanic white	13,379	69.2 (65.9, 72.4)	3,079	24.7 (23.3, 26.2)**	8,420	60.5 (58.4, 62.6)**	4,959	39.5 (37.4, 41.6)**
Non-Hispanic black	8,048	12.1 (10.3, 14.2)	2,112	25.0 (23.2, 26.8)	6,607	79.6 (77.7, 81.3)	1,441	20.4 (18.7, 22.3)
Mexican American	7,994	9.2 (7.8, 10.9)	4,848	64.6 (61.7, 67.4)	6,598	80.8 (78.8, 82.7)	1,396	19.2 (17.3, 21.2)
Age group, year								
<1	1,229	1.0 (1.0, 1.1)	65	3.9 (2.8, 5.5)	1,228	100 (99.7, 100)	1	0
1–3	2,676	4.1 (3.9, 4.4)	843	27.5 (25.1, 30.1)	2,135	73.4 (71.2, 75.5)	541	26.6 (24.5, 28.8)
4–8	3,137	6.9 (6.5, 7.4)	1,392	40.0 (36.8, 43.3)	2,227	63.4 (59.7, 67.0)	910	36.6 (33.0, 40.3)
9–13	3,740	7.3 (6.8, 7.7)	1,688	39.7 (37.0, 42.5)	3,075	77.3 (74.6, 79.9)	665	22.7 (20.1, 25.4)
14–18	4,413	7.5 (6.9, 8.0)	1,839	34.3 (31.9, 36.8)	3,860	82.7 (80.1, 85.1)	553	17.3 (14.9, 19.9)
19–30	3,637	15.9 (15.0, 16.9)	1,406	33.2 (30.6, 35.8)	2,848	73.4 (70.9, 75.8)	789	26.6 (24.2, 29.1)
31–50	5,599	28.9 (27.8, 29.9)	1,975	30.5 (28.4, 32.6)	3,896	65.4 (63.0, 67.6)	1,703	34.6 (32.4, 37.0)
51–70	5,104	20.5 (19.3, 21.8)	1,307	21.2 (19.0, 23.7)	3,066	52.8 (49.8, 55.8)	2,038	47.2 (44.2, 50.2)
>70	3,055	7.9 (7.3, 8.5)	446	11.4 (9.8, 13.4)	1,717	53.3 (50.9, 55.8)	1,338	46.7 (44.2, 49.1)
Reported folic acid supplement use^d^	8,538	34.2 (32.7, 35.6)	2,537	32.2 (30.4, 34.0)	–	–	–	–
Reported consumption of corn masa flour^e^	10,961	28.7 (27.3, 30.1)	–	–	8,424	29.6 (27.9, 31.3)	2,537	27.1 (25.5, 28.7)

a Percentages are column percents; weighted percentages.

b Percentages are row percents; weighted percentages.

c Race/ethnicity subanalyses were restricted to non-Hispanic whites, non-Hispanic blacks, and Mexican Americans.

d Reported folic acid supplement use is defined as consuming a supplement containing folic acid in the past 30 d.

e Reported consumption of corn masa flour is defined as consuming products that could contain corn masa flour on either day 1 or day 2 of the survey.

* Significantly different by sex, *P<*0.05 (Pearson chi-square test).

** Significantly different by race/ethnicity, *P*<0.05 (Pearson chi-square test).

Table 2 presents the percentage of US adults (aged≥19 years) with usual daily total folic acid intake exceeding the UL (1,000 µg). Including fortification of corn masa flour with folic acid, percentage point increases ranged from 0 to 0.5 with no statistically significant increases in the percentage of US adults who exceeded the UL, regardless of supplement use. No individuals who were supplement non-users exceeded the UL for total folic acid, with or without fortification of corn masa flour. Among adults, only those who reported consuming a supplement had usual daily intake exceeding the UL for total folic acid. The percentages of Mexican American adults who exceeded the UL stratified by sex, age, and acculturation factors are available in Supplemental Table 1 (See Supplemental file: Online Supplemental Material_Hamner, Supplemental Table 1). Findings were similar to the total adult US population. However, there was one stratum (Mexican Americans aged 31–50 years) that had a statistically significant increase in the percentage exceeding the UL (current: 0.3%; modeled: 0.6%, *P*=0.012). This age group had the lowest percentage exceeding the UL under the current scenario.

**Table 2:** Percentage of US population aged ≥19 year and those who report consuming corn masa flour, overall and stratified by folic acid supplement use, exceeding the tolerable upper intake level (UL)^a^ for usual total folic acid intake without (current) and with (modeled) folic acid fortification of corn masa flour by sex, race/ethnicity, and age, NHANES 2001–2008^b^,^c^

	Total			Non-supplement users			Supplement users		
			
	% ≥1,000 µg of total folic acid/day (95% CI)			% ≥1,000 µg of total folic acid/day (95% CI)			% ≥1,000 µg of total folic acid/day (95% CI)		
			
	Unweighted *n*	Current usual intake	Modeled usual intake	Unweighted *n*	Current usual intake	Modeled usual intake	Unweighted *n*	Current usual intake	Modeled usual intake
Total	1,7395	2.5 (1.6, 3.3)	2.6 (1.6, 3.5)	11,527	0	0	5,868	8.3 (5.4, 11.2)	8.6 (5.4, 11.7)
Sex									
Males	8,675	2.0 (1.6, 2.4)	2.1 (1.7, 2.4)	6,026	0	0	2,649	7.5 (5.2, 9.7)	7.7 (5.3, 10.1)
Females	8,720	2.9 (2.0, 3.8)	3.0 (2.2, 3.9)	5,501	0	0	3,219	8.9 (6.8, 11.1)	9.2 (7.0, 11.3)
Race/ethnicity^d^									
Non-Hispanic white	8,829	3.3 (2.7, 4.0)	3.5 (2.8, 4.1)	5,055	0	0	3,774	8.8 (6.1, 11.4)	9.0 (6.2, 11.7)
Non-Hispanic black	3,634	0.7 (0.2, 1.2)	0.8 (0.4, 1.1)	2,745	0	0	889	5.0 (2.9, 7.1)	5.0 (3.0, 7.0)
Mexican American	3,373	0.6 (0.3, 0.9)	0.8 (0, 1.9)	2,611	0	0	762	6.2 (3.8, 8.5)	6.5 (4.7, 8.3)
Age group, year									
19–30	3,637	1.2 (0.8, 1.7)	1.4 (0.9, 1.8)	2,848	0	0	789	8.9 (2.6, 15.2)	9.4 (1.9, 16.9)
31–50	5,599	1.9 (1.0, 2.7)	2.0 (1.1, 2.9)	3,896	0	0	1,703	NE^e^,^f^	NE^e^,^f^
51–70	5,104	3.7 (1.9, 5.6)	3.8 (2.1, 5.6)	3,066	0	0	2,038	9.0 (6.8, 11.1)	9.1 (7.1, 11.1)
> 70	3,055	2.7 (1.9, 3.5)	2.7 (1.9, 3.5)	1,717	0	0	1,338	6.6 (4.3, 9.0)	6.6 (4.4, 8.8)
	US population aged≥19 year who report consuming corn masa flour								
	Total			Non-supplement users			Supplement users		
	% ≥1,000 µg of total folic acid/day (95% CI)			% ≥1,000 µg of total folic acid/day (95% CI)			% ≥1,000 µg of total folic acid/day (95% CI)		
	Unweighted *n*	Current usual intake	Modeled usual intake	Unweighted *n*	Current usual intake	Modeled usual intake	Unweighted *n*	Current usual intake	Modeled usual intake
Total	5,134	1.9 (0.7, 3.0)	2.1 (0.7, 3.5)	3,590	0	0	1,544	7.8 (4.7, 10.9)	8.5 (6.2, 10.7)
Sex									
Males	2,569	0.8 (0.4, 1.2)	0.9 (0.5, 1.3)	1,876	0	0	693	4.5 (1.5, 7.4)	5.4 (2.3, 8.5)
Females	2,565	2.7 (0.4, 5.0)	3.0 (0.7, 5.2)	1,714	0	0	851	9.8 (5.4, 14.1)	10.0 (0, 24.2)
Race/ethnicity^d^									
Non-Hispanic white	1,804	2.8 (1.0, 4.6)	5.3 (1.2, 5.4)	1,024	0	0	780	8.7 (4.2, 13.2)	9.4 (6.0, 12.8)
Non-Hispanic black	680	0.2 (0, 0.5)	0.2 (0, 0.4)	510	0	0	170	NE^e^,^f^	NE^e^,^f^
Mexican American	2,223	0.2 (0.1, 0.4)	0.4 (0, 0.8)	1,746	0	0	477	4.5 (2.6, 6.4)	5.2 (2.6, 7.8)
Age group, year									
19–30	1,406	0.4 (0.1, 0.6)	0.5 (0.2, 0.7)	1,141	0	0	265	5.7 (0, 13.4)	6.6 (0, 22.4)
31–50	1,975	1.1 (0, 2.1)	1.2 (0.4, 2.0)	1,382	0	0	593	5.6 (2.7, 8.6)	6.4 (3.7, 9.1)
51–70	1,307	4.3 (0, 9.2)	4.8 (0, 9.7)	790	0	0	517	10.5 (8.3, 16.6)	11.1 (4.2, 18.0)
>70	446	0.3 (0, 0.6)	0.3 (0, 0.7)	277	0	0	169	1.5 (0, 4.4)	1.6 (0, 4.7)

a UL defined as ≥1,000 µg total folic acid.

b Data are adjusted for intake day of the week and interview method (in person or by telephone).

c All *P*-values comparing current and modeled percent over the UL were not statistically significant (*t*-test, *P*>0.05).

d Race/ethnicity subanalyses were restricted to non-Hispanic whites, non-Hispanic blacks, and Mexican Americans.

e NE=not estimable.

f NE due to instability in the measurement error model or degrees of freedom<12.

Table 2 also presents the percentage of adult corn masa flour consumers with usual daily intake exceeding the UL for total folic acid. Similar to the entire US population, with folic acid fortification of corn masa flour percentage point increases ranged from 0 to 2.5 with no statistically significant increases in the percentage who were exceeding the UL among adult corn masa flour consumers.

Table 3 presents the percentage of children aged 1–18 years who exceeded their age-specific UL. Under the current scenario, children aged 1–8 years, and specifically those who reported consuming supplements, were the most likely to have exceeded their age-specific UL. With folic acid fortification of corn masa flour, we estimated that these percentages would increase slightly ranging from 0.1 to 2.0 percentage points but increases were not statistically significant regardless of supplement use.

**Table 3:** Percentage of US population aged 1–18 year and those who report consuming corn masa flour, overall and stratified by folic acid supplement use, exceeding the tolerable upper intake level (UL) for usual total folic acid intake without (current) and with (modeled) folic acid fortification of corn masa flour by age, NHANES 2001–2008^a^,^b^

	% ≥age specific UL^c^ of total folic acid/day (95% CI)		
	
	Unweighted *n*	Current usual intake	Modeled usual intake
Age group, year			
Total population			
1–3	2,676	20.7 (18.4, 23.0)	21.3 (13.0, 29.7)
4–8	3,137	25.0 (14.7, 35.3)	26.9 (15.1, 38.6)
9–13	3,740	6.2 (0.9, 11.5)	6.6 (1.3, 12.0)
14–18	4,413	1.3 (0.6, 2.0)	1.5 (0.9, 2.2)
Non-supplement users			
1–3	2,135	3.2 (1.8, 4.7)	4.0 (2.7, 5.3)
4–8	2,227	3.4 (0, 6.9)	4.9 (0.4, 9.5)
9–13	3,075	0.2 (0, 0.5)	0.4 (0, 1.1)
14–18	3,860	0.2 (0.1, 0.4)	0.3 (0, 0.6)
Supplement users			
1–3	541	68.0 (53.6, 82.4)	69.2 (58.1, 80.4)
4–8	910	59.6 (37.9, 81.4)	61.6 (40.0, 83.1)
9–13	665	27.1 (13.1, 41.1)	28.7 (15.7, 41.7)
14–18	553	10.4 (3.9, 16.9)	11.1 (4.7, 17.5)
	US population aged 1–18 year who report consuming corn masa flour		
	% ≥age specific UL^c^ of total folic acid/day (95% CI)		
	Unweighted *n*	Current usual intake	Modeled usual intake
Age group, year			
Total population			
1–3	843	24.7 (7.5, 41.8)	28.4 (15.4, 41.3)
4–8	1,392	24.9 (8.1, 41.7)	29.3 (10.0, 48.6)
9–13	1,688	2.3 (1.2, 3.5)	3.3 (1.0, 5.5)
14–18	1,839	1.0 (0.2, 1.8)	1.3 (0.3, 2.3)
Non-supplement users			
1–3	662	4.0 (0, 2.2)	6.4 (3.4, 9.5)
4–8	1,059	3.4 (0, 5.2)	6.5 (0, 13.6)
9–13	1,411	0.2 (0, 0.4)	0.3 (0, 0.6)
14–18	1,637	0.2 (0, 0.4)	0.3 (0, 0.6)
Supplement users			
1–3	181	72.9 (54.1, 91.6)	76.3 (59.1, 93.5)
4–8	333	66.1 (42.9, 89.2)	70.7 (44.1, 97.4)
9–13	277	17.1 (0.2, 34.0)	21.9 (5.2, 38.6)
14–18	202	9.1 (0, 21.5)	11.2 (0, 27.8)

a Data are adjusted for intake day of the week and interview method (in person or by telephone).

b All *P*-values comparing current and modeled percent over the UL were not statistically significant (*t*-test, *P*>0.05).

c UL defined by Institute of Medicine age groups: 1 to 3 y=300 µg; 4 to 8 y=400 µg; 9 to 13 y=600 µg; 14 to 18 y=800 µg.

The percentages of Mexican American children who exceeded their age-specific UL stratified by age group are available in Supplemental Table 2 (See Supplemental file: Online Supplemental Material_Hamner, Supplemental Table 2). Overall, there were no statistically significant increases in the percentage of Mexican American children exceeding their age-specific UL with fortification of corn masa flour.

Table 3 also presents the percentage of corn masa flour consumers aged 1–18 years who exceeded their age-specific UL. Children aged 1–8 years were the most likely to exceed their age-specific UL under both scenarios. Regardless of age or supplement use, there were no statistically significant increases in the percentage exceeding their age-specific UL with fortification of corn masa flour and percentage point increases ranged from 0.1 to 4.6.

## Discussion

Modeling has shown that fortification of corn masa flour with folic acid would increase folic acid intake among the US population, specifically targeting Mexican Americans (11, 30). We found that fortification of corn masa flour would not increase the percentage of individuals exceeding the UL for total folic acid. Our data indicate that supplement use was the main factor related to exceeding the UL, regardless of stratification by sex, race/ethnicity, age group, or reported consumption of corn masa flour. Although these findings must be considered within the context of supplement use, our data indicate that only one third of the US population and corn masa flour consumers reported consuming a supplement containing folic acid at some point in the past 30 days. In addition, data from NHANES 2003–2006 indicate that adults who report consuming >400 µg of folic acid from supplements were the ones who were exceeding the UL (31).

Recently, there has been a call to consider fortification of corn masa flour with folic acid as an approach to target Hispanics and reduce the racial/ethnic disparity in NTD prevalence (32). Results from a modeling exercise assessing the potential impact of folic acid fortification of corn masa flour suggest that this could be an effective way to target Mexican Americans (11). Additionally, updated results demonstrate that folic acid fortification of corn masa flour could target a high-risk population, namely, lower acculturated Mexican American women (30). Given that fortification is an intervention that impacts the entire population, policy makers must take into account the safety of the intervention, including whether or not individuals would consume higher amounts of folic acid as a result. The UL has been a generally accepted level to assess this concern (1). The results of our analysis suggest that fortification of corn masa flour would not significantly change the current percentage of individuals exceeding the UL. Additionally, our results indicate that supplement use, not corn masa flour fortification, would continue to be the driving force behind the percentage exceeding the UL among adults, even if corn masa flour were fortified. These observations are consistent with what has been reported elsewhere in the US and in Canada (7, 31, 33–35).

Among children aged 1–18 years, our results also indicate that fortification of corn masa flour would not lead to a larger percentage exceeding the UL for folic acid. Similar to adults, the primary factor related to exceeding the UL among children is supplement use. The situation for children is slightly different from adults in that a small percentage of children who do not use supplements also exceed their age-specific UL, which is due to the lower UL values set for children. Although these values are the published values from the IOM and must be recognized, some have questioned whether or not they are clinically meaningful for children (10, 7).

The issue of children and high folic acid intake levels has been considered outside the US as other countries have assessed whether or not they should implement mandatory fortification with folic acid. For example, in 2007 the Food Standards Australia New Zealand report determined that children's intakes were within the five-fold safety margin established for adults and that ‘the proportion of children exceeding the UL did not pose any risk to their health’ (36). The United Kingdom did not set a UL for children; setting instead a ‘Guidance Level’ (37). The Guidance Level is an approximate indication of intake that would not be expected to cause adverse effects and is set when there are insufficient data to determine a safe upper level of a micronutrient (37). A safe upper level for folic acid intake was not set in the United Kingdom for either children or adults because the available evidence on adverse effects of folic acid was not considered to be sufficiently robust. A Guidance Level of 1,000 µg folic acid/day among adults was set based on concerns that intake above this level might mask signs of vitamin B12 deficiency (37). Guidance Levels were not set for children, as there were no available data reporting adverse effects in children (4).

In addition to questions about the UL's clinical relevance for children, there are also questions about its value for adults in an era with more advanced biomarker assessment. Clinical recommendations for diagnosing a vitamin B12 deficiency are more thorough since the publication of the IOM's report on folic acid in 1998. If vitamin B12 deficiency is assessed using a complete blood count and smear only, it could be masked by very high folic acid intake (1). However, when vitamin B12 deficiency is diagnosed using a combination of history, signs and symptoms, a complete blood count with smear, and an assessment of serum vitamin B12 and serum folate blood concentrations with the potential to follow-up with an assessment of methylmalonic acid concentrations (38), much or all of the current concern regarding a potential for masking should be alleviated. Given the low prevalence of vitamin B12 deficiency among children [defined as serum vitamin B12 concentrations <200 pg/mL, less than 0.5% of US children <19 years were deficient in 2003–2006 (7)] and the ability to determine vitamin B12 deficiency based on multiple assessment methods (38), these issues could be considered during future discussions of the Dietary Reference Intake values for folic acid. Regardless of potential future changes, fortification of corn masa flour in our model does not significantly change the percentage of adults or children exceeding the currently established UL.

There were several limitations to these analyses. First, NHANES is a cross-sectional study and cannot be used to determine causation and is limited to associations only; we are not able to draw any conclusions about benefits and safety of folic acid using these data. Second, the dietary analyses using NHANES data are subject to the limitations of the Food and Nutrient Database for Dietary Studies and could over-or underestimate the amount of folic acid in some foods. Third, estimates of folic acid from dietary supplements might not truly represent daily intake among individuals who infrequently use dietary supplements and could have under-estimated the within person variation (31). Fourth, analyses reported by race/ethnicity were limited to non-Hispanic whites, non-Hispanic blacks, and Mexican Americans. Estimates were only generalizable to Mexicans Americans and were not available for other Hispanic ethnicities; however, individuals who reported being of a different Hispanic ethnicity were included in analyses reported for the total population and by sex and age. Fifth, PC-SIDE was used to estimate the percentage of individuals who were exceeding their age-specific ULs. For many of these estimates, the set point was at the tail of the distribution (i.e. 1,000 µg of total folic acid) and because of the instability of estimates at these tails, these percentages should be interpreted with caution (39). Lastly, due to NHANES data being specific to the US only, we are unable to extrapolate our results to other countries. However, our methods for modeling the impact of fortification on folic acid intake using data from a national nutrition survey could be applied to data from other countries for which fortification is being considered. Limitations regarding the modeling of corn masa flour fortification have been described elsewhere (11, 30). There are also several strengths to this study. NHANES provides data on a representative sample of the US population and several subgroups, including Mexican Americans. It also contains two days of dietary intake data, which allow us to estimate an individual's usual intake and determine the percentage above or below specific cut-off points.

## Conclusions

Fortification of corn masa flour does not significantly change the percentage of adults or children exceeding the UL. Given that folic acid fortification of corn masa flour would selectively target a public health disparity in folic acid intake among Mexican Americans and not increase the percentage of the total US population including subgroups exceeding the UL, concerns regarding intake above the UL are better directed at dietary supplements, which we identified as the primary factor in folic acid intake above the UL.

## Appendix 1

Foods considered to contain corn masa flour, NHANES 2001–2008^a^

**Table:**

TORTILLA, CORN
TACO SHELL, CORN
CRACKER CORN (INCLUDES STONED CORN CRACKER)
SALTY SNACKS, CORN/CORNMEAL BASE, NUT /NUG, TOASTED
SALTY SNACKS, CORN OR CORNMEAL, CORN CHIPS, CHEESE
SALTY SNACKS, CORN OR CORNMEAL, CORN PUFFS, TWISTS
SALTY SNACKS, CORN OR CORNMEAL, TORTILLA CHIPS
SALTY SNACKS, CORN/CORN-CHEESE CHIPS, UNSALTED
SALTY SNACKS, CORN/CORNMEAL BASE, TORTILLA CHIPS LIGHT
SALTY SNACKS, TORTILLA CHIPS, FAT FREE, W/ OLEAN
SALTY SNACKS, CORN/CORNMEAL BASE, TORTILLA, LOWFAT, BAKED
SALTY SNACKS, CORN/CORNMEAL, TORTILLA, LOWFAT, BAKED, NO SALT
SALTY SNACKS, CORN/CORNML BASE,W/OAT BRAN, TORTILLA CHIPS
SALTY SNACKS, CORN BASED/CHEESE PUFFS & TWISTS, LOWFAT
TORTILLA CHIPS, UNSALTED
CORN FLOUR PATTIES OR TARTS, FRIED
NACHOS W/ BEEF, BEANS, CHEESE & SOUR CREAM
NACHOS W/ CHEESE & SOUR CREAM
NACHOS W/ CHEESE, MEATLESS, NO BEANS
NACHOS W/ BEANS, NO CHEESE
NACHOS W/ BEANS & CHEESE
NACHOS W/ BEEF, BEANS & CHEESE
NACHOS W/ BEEF & CHEESE
NACHOS W/ CHILI
NACHOS W/BEEF, BEANS, CHEESE, TOMATOES & ONIONS
NACHOS WITH CHICKEN/TURKEY & CHEESE
ENCHILADA W/ BEEF, NO BEANS
ENCHILADA W/ BEEF & BEANS (INCLUDE ENCHILADA, NOT FURTHER SPECIFIED)
ENCHILADA W/ BEEF, BEANS, & CHEESE
ENCHILADA W/ BEEF & CHEESE, NO BEANS
ENCHILADA W/ HAM & CHEESE, W/O BEANS
ENCHILADA W/ CHICKEN, TOMATO-BASE SAUCE
ENCHILADA W/ CHICKEN & BEANS, TOMATO-BASE SAUCE
ENCHILADA W/ CHICKEN, BEANS & CHEESE, TOMATO SAUCE
ENCHILADA W/ CHICKEN & CHEESE, NO BEANS, TOMATO SAUCE
ENCHILADA W/ BEANS, MEATLESS
ENCHILADA W/ BEANS & CHEESE, MEATLESS
ENCHILADA W/ CHEESE, MEATLESS, NO BEANS
ENCHILADA W/ SEAFOOD, TOMATO SAUCE
BEEF ENCHILADA DINNER, NOT FURTHER SPECIFIED (FROZEN MEAL)
BEEF ENCHILADA, GRAVY, RICE, REFRIED BEANS (FROZEN)
CHEESE ENCHILADA W/ BEANS & RICE (FROZEN MEAL)
CHEESE ENCHILADA (FROZEN MEAL)
CHICKEN ENCHILADA ( DIET FROZEN MEAL)
CHICKEN ENCHILADA W/SALSA, RICE, VEG, DES (DIET FROZEN)
CHILAQUILES, TORTILLA CASSEROLE W/ SALSA, CHEESE, EGG
CHILAQUILES, TORTILLA CASSEROLE, NO EGG
POCHITO (FRANKFURTER/HOT DOG & BEEF CHILI IN TORTILLA)
HUEVOS RANCHEROS
MEXICAN CASSEROLE W/ BEEF & BEANS
MEXICAN CASSEROLE W/ BEEF (INCLUDES FRITO PIE, NOT FURTHER SPECIFIED)
SOPA DE TORTILLA, MEXICAN STYLE TORTILLA SOUP
TAMALE W/ MEAT &/OR POULTRY (INCLUDES TAMALE, NOT FURTHER SPECIFIED)
TAMALE, MEATLESS, CARIBBEAN OR PUERTO RICAN STYLE
TAMALE, PLAIN, MEATLESS, NO SAUCE, MEXICAN
TAMALE CASSEROLE W/ MEAT
TAMALE CASSEROLE, PUERTO RICAN (TAMALES EN CAZUELA)
TAMAL IN A LEAF, PUETRO RICAN (TAMALES EN HOJA)
TAMALE, SWEET
TAMALE, SWEET, W/ FRUIT
TAMALE WITH MEAT
TAMALE WITH CHICKEN
TAMALE, PLAIN, MEATLESS, NO SAUCE, PUERTO RICAN STYLE
PUPUSA, CHEESE-FILLED
PUPUSA, MEAT-FILLED
PUPUSA, BEAN-FILLED
CHALUPAS W/ BEANS, CHEESE, LETTUCE AND TOMATO
CHALUPA W/ BEEF, CHEESE, LETTUCE, TOMATO & SOUR CREAM
CHALUPA W/ BEEF, CHEESE, LETTUCE, TOMATO, SALSA
CHALUPAS W/ BEANS, CHICKEN & CHEESE
CHALUPA W/ CHICKEN, CHEESE, LETTUCE, TOMATO & SOUR CREAM
CHALUPA W/ CHICKEN, CHEESE, LETTUCE, TOMATO, SALSA
GORDITA/SOPE SHELL, PLAIN NO FILLING, GRILL, NO FAT ADDED
GORDITA/SOPE SHELL, PLAIN, NO FILLING, FRIED IN OIL
QUESADILLA W/ CHEESE, MEATLESS
QUESADILLA W/ MEAT & CHEESE
QUESADILLA W/ POULTRY & CHEESE
TACO/TOSTADA W/ BEEF, CHEESE, LETTUCE
TACO OR TOSTADA W/ BEEF, LETTUCE, TOMATO & SALSA
TACO/TOSTADA W/ BEEF, CHEESE, LETTUCE, TOMATO AND SALSA
TACO W/BEEF, CHEESE, LETTUCE, TOMATO, SOUR CREAM
SOFT TACO W/ BEEF, CHEESE, & LETTUCE (INCLUDES TACO BELL)
SOFT TACO W/ CHICKEN, CHEESE & LETTUCE
SOFT TACO W/ CHICKEN, CHEESE, LETTUCE, TOMATO & SOUR CREAM
TACO/TOSTADA W/ CHICKEN/TURKEY, LETTUCE, TOMATO, SALSA
SOFT TACO W/ BEEF, CHEESE, LETTUCE, TOMATO, SALSA
SOFT TACO WITH BEAN, CHEESE, AND LETTUCE
SOFT TACO WITH BEAN, CHEESE, LETTUCE, TOMATO &/OR SALSA
SOFT TACO W/BEAN, CHEESE, LETTUCE, TOMATO &/OR SALSA, SOUR CREAM
TACO OR TOSTADA W/ FISH
TACO/TOSTADA W/ CHICKEN, CHEESE, LETTUCE, TOMATO, SALSA
TACO/TOSTADA W/ BEANS, MEATLESS, LETTUCE, TOMATO, SALSA
TACO/TOSTADA W/ BEANS, CHEESE, LETTUCE, TOMATO, SALSA
TACO OR TOSTADA W/ BEANS, CHEESE, MEAT, LETTUCE, TOMATO, SALSA
TACO SALAD W/ BEEF & CHEESE, CORN CHIPS
FLAUTA, NOT FURTHER SPECIFIED
FLAUTA W/ BEEF
FLAUTA W/ CHICKEN
TAQUITOES
TAQUITOS WITH MEAT
TAQUITOS WITH CHICKEN
TACO W/ CRAB MEAT, PUERTO RICAN (TACOS DE JUEYES)
ATOLE (CORNMEAL BEVERAGE)

a Foods are listed in the order they are found in NHANES food codes.
